# Clinical Trials and Outcome Measures in Adults With Hearing Loss

**DOI:** 10.3389/fpsyg.2021.733060

**Published:** 2021-11-05

**Authors:** Kevin J. Munro, William M. Whitmer, Antje Heinrich

**Affiliations:** ^1^Manchester Centre for Audiology and Deafness, School of Health Sciences, University of Manchester, Manchester, United Kingdom; ^2^Manchester Academic Health Science Centre, Manchester University Hospitals NNS Foundation Trust, Manchester, United Kingdom; ^3^Hearing Sciences–Scottish Section, Division of Clinical Neuroscience, University of Nottingham, Glasgow, United Kingdom; ^4^Institute of Health and Wellbeing, University of Glasgow, Glasgow, United Kingdom

**Keywords:** clinical trials, outcome measures, minimal important difference, interventions, hearing loss, hearing-related outcomes, clinically meaningful

## Abstract

Clinical trials are designed to evaluate interventions that prevent, diagnose or treat a health condition and provide the evidence base for improving practice in health care. Many health professionals, including those working within or allied to hearing health, are expected to conduct or contribute to clinical trials. Recent systematic reviews of clinical trials reveal a dearth of high quality evidence in almost all areas of hearing health practice. By providing an overview of important steps and considerations concerning the design, analysis and conduct of trials, this article aims to give guidance to hearing health professionals about the key elements that define the quality of a trial. The article starts out by situating clinical trials within the greater scope of clinical evidence, then discusses the elements of a PICO-style research question. Subsequently, various methodological considerations are discussed including design, randomization, blinding, and outcome measures. Because the literature on outcome measures within hearing health is as confusing as it is voluminous, particular focus is given to discussing how hearing-related outcome measures affect clinical trials. This focus encompasses how the choice of measurement instrument(s) affects interpretation, how the accuracy of a measure can be estimated, how this affects the interpretation of results, and if differences are statistically, perceptually and/or clinically meaningful to the target population, people with hearing loss.

## Introduction

Clinical trials are a type of research that study health interventions and evaluate their effects on human health outcomes (World Health Organisation, 2018). James Lind is credited for conducting the first clinical trial in humans (see for example, Collier, 2009). In 1747, Lind investigated different treatments for scurvy. He demonstrated that, in sailors living under the same conditions, it was only those who were provided with fruit (specifically, Vitamin C) that recovered. The purpose of the intervention in a clinical trial might be to prevent, diagnose or, in the case of Lind, treat a health condition. The conduct and quality of clinical trials is critical since they provide the evidence base for improving practice in health care. Many health professionals, including those working within or allied to hearing health, are expected to conduct or contribute to clinical trials.

Grading of Recommendations, Assessment, Development, and Evaluations (GRADE; Atkins et al., 2004) is a framework commonly used to assess quality of evidence based on study limitations, inconsistency, indirectness, imprecision, and publication bias, e.g., outcomes from non-randomized studies without blinding would be considered low. As with many areas of healthcare, systematic reviews in hearing science and audiology have highlighted a dearth of good quality clinical trials. Notable in this context are reviews published by the National Institute for Health and Care Excellence (NICE), a public body sponsored by the United Kingdom government that provides evidence to improve health and social care, and the Cochrane Database of Systematic Reviews (CDSR), the leading journal and database for systematic reviews in health care:

1. NICE published reviews as part of national guidelines (NG) on assessment and management of adult hearing loss (NG98; National Institute for Health and Care Excellence, 2018), and assessment and management of tinnitus (NG155; National Institute for Health and Care Excellence, 2020). Both guidelines include around 20 systematic reviews on areas of uncertainty or variation in clinical practice. 50–60% of the systematic reviews revealed no evidence on which to base clinical recommendations. The remaining 40–50% of the systematic reviews identified supporting evidence; however, the quality of the individual studies was mostly graded as low due to risk of bias (see later).
2. CDSR published a review of the effects of hearing aids in everyday life for people with mild to moderate hearing loss. This revealed benefits; however, the evidence was based on five studies, and their quality was graded as moderate (Ferguson et al., 2017).

The current article redresses the limited evidence base by providing an overview of the design, analysis and conduct of clinical trials. Judicious use of selected studies highlight potential methodological limitations as well as examples of good practice. The aim is to provide guidance to hearing health professionals about the key elements that define the quality of a trial. Detailed information is provided on outcome measures, and on how hearing-related outcome measures affect clinical trials: (i) how the choice of measurement instrument(s) affects interpretation, (ii) how the accuracy of a measure can be estimated, (iii) how this affects the interpretation of results, and (iv) if differences are statistically, perceptually and/or clinically meaningful to the target population, people with hearing loss.

## Identifying the Research Question

Before designing a clinical trial, an essential starting point is to craft a carefully worded research question. Evidence-based medicine provides an explicit framework for formulating research questions that can be used when: (i) designing clinical trials or (ii) searching the literature for studies to be included in a systematic review of the literature. The four components of the question are contained in the PICO mnemonic: Population (P), Intervention (I), Comparator (C), and Outcome (O).

An example of a research question in the PICO format would be, “What is the clinical- and cost-effectiveness [outcome] of monitoring and follow-up regimes [intervention] for adults offered NHS hearing aids for the first time [population], compared to usual care [comparator]. The same approach was used by NICE when preparing the clinical guidelines mentioned earlier.

## The Hierarchy of Evidence

Hierarchies of evidence, developed to aid the interpretation and evaluation of research findings, are a core principal of Evidence-Based Practice (EBP). They rank research according to its validity, and in particular, risk of bias. While many research study designs exist (e.g., cohort, case-controlled, cross-sectional and case series/reports), well conducted randomized controlled trials (RCT) are generally considered the gold standard because they provide the lowest risk of bias and, hence, the highest quality of evidence. The first step to building high-quality evidence for clinical practice should always be a recent well-conducted systematic review following a standardized reporting method such as the Preferred Reporting Items for Systematic Reviews (PRISMA).^1^ An alternative design to a RCT is an observational study, so called because the researcher observes individuals without manipulation or intervention. These can be useful in instances where RCTs are not appropriate. For example, the effectiveness of parachutes has not been proven in a RCT where participants are randomized to parachute or placebo (Smith and Pell, 2003). In this example, the effect size would be very large because death and serious trauma is much more likely in the placebo group. However, when effect sizes are smaller (which applies to the vast majority of questions), confounds and bias may distort the effect size. In such cases, all efforts should be made to set up an RCT. To appreciate the potential disadvantages of observational designs compared to a RCT trial, consider the following study by Noble and Gatehouse (2006). They used an observational design to compare existing adult hearing aid users of bilateral or unilateral hearing aids. Their results showed that bilateral hearing aids offer advantages in demanding and dynamic listening situations that were not conferred by unilateral hearing aids. However, due to the design it is not possible to know if the natural selection of groups introduced a bias and led to a miss-estimation of the effect.

Systematic errors have the potential to result in the wrong conclusions about the effects of the intervention. The risk of systematic errors differs between designs and is more likely for observational designs than RCT. Two types of systematic errors are biases and confounds. An example of an experimental confound is age. If, for example, a higher proportion of older people receive the intervention than the control, age-related differences, unrelated to the intervention, could affect the results. An example of bias is when researchers *or* participants expect the new intervention to generate a better outcome. For example, Dawes et al. (2011, 2013) examined the effect of participant expectation when comparing two hearing aids that were identical except one was labeled “new” and the other “conventional.” Mean performance with the hearing aid labeled “new” was significantly higher on all outcome measures. These studies demonstrate that placebo effects can, and do, affect hearing aid trials. Initial preferences can dominate outcomes, as shown in hearing-aid RCTs investigating unilateral and bilateral fittings. For example, Cox et al. (2011) showed that 80% of participants could be predicted based on initial preference for one or two hearing aids. Additionally, Naylor et al. (2015) demonstrated that the outcome for the same technology was influenced by how involved the participant was in the fitting process. Therefore, measuring preferences and attitudes related to the intervention should be included to help control for such confounds in the analysis. Another set of biases are performance and detection biases when systematic differences exist between groups in terms of care and measurement of outcomes, which can be minimized through blinding. By reducing the risk of confounds and bias, any difference in outcome at the end of the trial can be more robustly attributed to the intervention.

Clinical trials in humans are commonly classified into four types or “phases,” depending on their aim. Within a trial, there are typically four stages to its preparation and operation: pre-trial, trial set-up, during trial and end of trial. Table 1 details the phases and gives examples of activities carried out at each stage of any clinical trial. Hackshaw (2009) provides a comprehensive overview of the design, conduct and analysis of trials, ideal for busy health professionals who read or undertake clinical research.

**TABLE 1:** Phases (types) of clinical trials and examples of key activities in each clinical trial process.

Phase	Explanation
One	An *exploratory* investigation of safety and the effects of dosage in a small number of healthy participants.
Two	A *preliminary* estimate of efficacy (i.e., the potential of the intervention to provide benefit), in a small number of participants with the specific health condition.
Three	A *definitive* trial of effectiveness, involving a relatively large number of participants who are randomized to the intervention(s) or control.
Four	*Monitors* side effects and how well the intervention works over a longer period and in a very large number of participants.

**Stage**	**Key activities**

Pre-trial	• Formulate the PICO research question • Design trial
Trial set-up	• Protocol and ethics • Operating procedures, including case report forms for collecting de-identified data • Set-up site(s) • Register trial
The trial	• Collect and store data • Regularly review for protocol adherence
End of trial	• Lock database and undertake statistical analysis • Identify and deal with missing data • Disseminate trial findings

## Methodological Considerations

In order to ensure that clinical trials are executed well, some key methodological issues need to be considered. These include design, randomization, and blinding; all three pose particular challenges to running a hearing-specific RCT.

### Design

**FIGURE 1:**
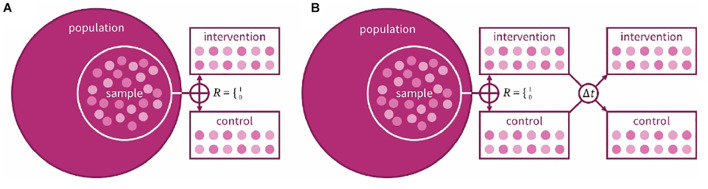
Schematic example of a **(A)** parallel and **(B)** crossover design for a two-arm clinical trial. In both designs, a sample is taken from the target population, randomly allocated to intervention or control group. In the crossover design, the same participants are then given the other intervention after an interim washout period (At).

A cardinal decision in every clinical trial is the choice of design. Fundamentally, the research team has the decision between two designs: a crossover design where participants receive all interventions in a randomized (or counter-balanced) order, or a parallel-group design where participants are randomized to a single intervention (Figure 1). An advantage of the crossover design is that it is a within-groups design: each participant acts as their own control, increasing statistical power. In hearing studies, where the emphasis is usually less on cure and more on benefit and quality of life (QoL), our preference is judicious use of crossover trials. Marriage et al. (2004) used a crossover design when comparing three prescriptions for hearing aid gain settings; there was, however, an issue in its crossover design: tolerance for greater gain increased over the course of the trial regardless of intervention order. Hearing-aid studies using a crossover design often do not include washout periods (Arlinger et al., 2008; cf. Cox et al., 2016) which may reduce carryover effects from one intervention to the next. For hearing training and support interventions, crossover randomization would confound the effect of the intervention with its order (i.e., outcomes following a training period would not be expected to be equivalent to outcomes preceding a training period), hence parallel designs have been used (e.g., Meijerink et al., 2020). A parallel group design contains more natural variation, making it harder to know whether any variation in results is due to the intervention or differences between the participants in the groups. Humes et al., 2017 used a parallel design to study the efficacy of generically fit hearing aids vs. individually fit and placebo devices, randomly assigning participants to one of the three arms. The population to be tested also needs to be considered; in interventions with hearing-aid users, for example, halo effects may lead to greater effects for new compared to experienced users (Ivory et al., 2009).

### Randomization

In an RCT, a sample of participants from the population of interest are randomly allocated to receive the experimental or control/comparator intervention (the latter may be “usual care” or a placebo). The purpose of randomization is to reduce systematic differences in the characteristics of participants allocated to each group. In the case of Lind’s scurvy trial, the population of interest (sailors with scurvy) were randomly allocated to receive interventions including seawater, nutmeg and garlic and fruit. In hearing science, within-group crossover designs are much more common. The type of randomization can be critical to allocation and analyses of the trial (Lachin et al., 1988). For hearing-related RCTs the sample size is usually <200, so simple randomization could lead to imbalanced group sizes. When using multiple clinics or outcomes with known covariates, both common in hearing trials, stratified randomization is necessary to insure reasonably balanced allocation across sites and/or covariates. For example, Humes et al. (2017) first stratified participants by unaided speech-in-noise performance, an expected covariate with their outcomes, before then allocating each stratum randomly to a different arm. A newer randomization technique, called merged block (van der Pas, 2019), combines block and simple randomization while avoiding the biases of both, and could be well-suited to hearing RCTs.

### Blinding

Blinding is a critical methodological feature of RCTs that reduces the risks of confounds and biases. Ideally, blinding should extend to everyone associated with the trial including clinicians, data collectors and data analysts. Clinical trials are described as double-blinded if both the researcher and participant are unaware of treatment allocation. A single-blinded study usually means the participant is unaware which treatment has been allocated. Blinding is more difficult to incorporate in trials of medical devices and surgical interventions than trials of medical therapies, which usually include placebo medications. Cox et al. (2016) investigated the effect of basic vs. premium hearing-aid features on subjective outcomes in a single-blinded study with no statistically significant difference between feature levels. In theory, this could have been a double-blinded study if the researcher responsible for data collection and analysis was also blinded from the hearing aid prescription and fitting. For many studies involving standard hearing aids, the devices need to be individually fit, potentially unblinding the audiologist. The audiologist would then need to be outwith the research team and blind to the aims of the study. In the Cochrane systematic review evaluating the effects of hearing aids for mild-to-moderate hearing loss in adults (Ferguson et al., 2017), the risk of performance and detection bias was rated as high because blinding was inadequate or absent. More recently, there have been attempts to maintain blinding. The use of placebo hearing aids allows blinding if they are visibly identical to active hearing aids. Studies by Adrait et al. (2017) and Humes et al. (2017) both used placebo hearing aids that provided minimal gain. These studies demonstrate that it is possible to blind participants and outcome assessors in hearing aid trials where the amplification characteristics can be concealed. Also, in a double-blinded RCT investigating the effectiveness of sound therapy in people with a reduced audiometric dynamic range, Formby et al. (2015) used conventional and placebo-controlled sound generators where the output of the placebo decayed to silence after 1 h of use in the ear.

## Health-Related Outcome Measures

The most important question of any clinical trial is whether the trial’s intervention was successful. The question is answered by means of primary and secondary outcome measures. Primary outcome measures capture the most evident or most important changes connected to the intervention (Vetter and Mascha, 2017). Secondary outcome measures assess aspects of the intervention in finer detail, for example, in order to understand mechanisms of change.

Once it is clear what the main expected change is, the vital question is how to capture this change. Outcome measurements can be objective (physiological or behavioral) or subjective, generalized or specific and clinician- or patient-reported. Other important considerations are the period being measured, and the measures’ generalizability, reliability, and relevance.

### Objective Versus Subjective Outcomes

Some changes are only measurable by one type of outcome. One example is satisfaction, which can only be assessed as a subjective measure. However, subjective measures always need to be treated with caution. Satisfaction is a good example as the aforementioned study by Humes et al. (2017) found relatively good satisfaction with a placebo hearing aid.

For other outcomes, both objective and subjective measures exist. The combination of different instruments, such as objective and subjective measures of change in hearing ability, often will provide greater sensitivity and interpretability than a single measure. Further, using multiple measures will help counteract any dependence a single outcome has on participants or practitioners when blinding is an issue (e.g., the intervention difference cannot be concealed). However, the more outcome measures included in a trial, the greater the risk that results do not concur and potentially lead to opposing interpretations. One example is hearing aid use, which can differ between patients’ self-reported use and their devices’ data logging. For example, Solheim and Hickson (2017) reported a mean of 8.4 and 6.1 h for self-report and data logging, respectively. A possible alternative to measuring hearing aid use by data logging is to measure persistence through requests for supply of batteries (Zobay et al., 2021). Future measures may also be able to tap into usefulness – the desired outcome for which use and persistence are surrogates.

### Outcome Measurement Period

Deciding on the time point of assessment is particularly difficult, as it needs to include considerations of the temporal nature of the intervention. For hearing-aid trials, there may be an auditory acclimatization period before achieving full objective benefit (Dawes and Munro, 2017), whereas initial subjective benefit may decline over time (Humes et al., 2002). In addition, care must be taken to monitor the environments during the measurement period (e.g., via data logging) to ensure it is homogenous (Humes et al., 2018). For other studies, including training studies, the main interest might be in the time course and longevity of change. In the case of the latter, it needs to be carefully considered whether change is best assessed immediately after the intervention, or 6 weeks, 6 months or a year later. Wisely chosen test intervals may, for example, show whether training effects persist or weaken after the end of regular training (Henshaw et al., 2021).

### Generalizability of Outcomes

The question of generalizability reflects the tension between choosing standardized tools that are validated but have limited specificity to a particular health condition versus tools that are specific to a health condition but possibly newly created or modified, and often insufficiently validated. One example are QoL measures. As shown by Heinrich et al. (2015), a standardized generic QoL questionnaire such as the EQ-5D may not show any correlation with speech-in-noise performance, while a hearing-specific extension, the HR EQ-5D, does, but has not been appropriately standardized and validated. In the interest of building a body of evidence that can support CDSR and healthcare-system decisions (e.g., NICE) to improve clinical practice, some standardization and validation of outcomes measures will be essential. The Health Utility Index (HUI3) may provide a compromise as it is a standardized tool that has shown some sensitivity to hearing-aid provision (Barton et al., 2004).

A number of initiatives have been set up to understand what measurement instruments are being used within a field, how accurate, reliable and valid they are for what they aim to assess and how a core minimum outcome set could look like. Initiatives such as COMET (Core Outcome Measures in Effectiveness Trials)^2^ bring together research groups interested in the development and application of agreed standardized sets of outcomes that should be measured and reported as minimum core sets in all clinical trials of a specific condition. One hearing-aid related outcome measure that was developed in a consortium resembling (but prior to) COMET is the seven-item International Outcome Inventory for Hearing Aids (IOI-HA; Cox et al., 2000).

### Outcome Reliability

If the validity and reliability of an outcome measure are in doubt, any interpretation of the results may suffer. COSMIN (Consensus-based Standards for the selection of health Measurement INstruments)^3^ is an expert-led initiative that developed standards for the evaluation of health-status measures. Any outcome measure included in a trial should conform to their standards. A critical aspect of an outcome measure’s methodological quality is its retest reliability. There are various ways of calculating reliability estimates (see Heinrich and Knight, 2020 for a discussion). The broader point, however, is that the retest reliability for many hearing outcome measures is rather poor, leading to “non-trivial” minimum/critical differences required to show an effect of an intervention (Weinstein et al., 1986; Cox and Rivera, 1992). Retest reliability and critical differences are also rather poor for standard speech-in-noise tests (Heinrich and Knight, 2020), making it a challenge to use them as outcome measures for a hearing RCT in which small effects may be expected.

### Relevance of Outcomes

Statistical significance is only one aspect of change. Equally important is that changes are perceptually noticeable and clinically relevant. Often it is possible to show that a change is statistically significant, particularly on a group level, but not perceptually noticeable or meaningful for an individual (e.g., improvement in signal-to-noise ratio that was not perceived by the participants; McShefferty et al., 2015, 2016), hence may lack relevance for the patient. Relevance at the clinic level can be achieved from comparing results against a (minimal) clinically important difference [(M)CID], a stakeholder-defined threshold of the proportional alleviation of a dysfunction or reduction in its prevalence. As hearing-loss interventions are compensatory, not restorative, (M)CIDs can seem ill suited to measuring clinically important differences, though it is possible, as demonstrated by Skarżyński et al. (2018) for tinnitus improvement after middle ear surgery. By first defining the threshold for a successful intervention, abetted by using validated measures that have a no-change midpoint, it is possible to report the percentage in alleviation for a particular hearing problem.

## Reproducibility

The reproducibility of research is key to scientific advancement. It means that comparable results are obtained by methodologically closely matched but independent studies. Many fields, including biomedical science, suffer from a reproducibility crisis (de Vries et al., 2018) led by poor research practices and a well-established bias in scientific journals to preferentially publish novel and statistically significant findings which support the experimental hypothesis (Fanelli, 2012; Open Science Collaboration, 2015). Reproducibility can be increased in a number of ways, many of them applicable to clinical trials research. First, it is important to ensure that every phase of the research cycle is as transparent and open as possible, so that readers can fully evaluate the work. This research practice is referred to as “open science” (Kathawalla et al., 2021) and often contains the following three components: pre-registration, open data and open materials (Svirsky, 2019). Pre-registration makes information available in the public domain about the design and conduct of an intended study *before* collection of data (Munro and Prendergast, 2019). Open data and materials refers to depositing the datasets and test materials from the trial in the public domain. In addition to adhering to open science principles, the robustness of results are further bolstered by conducting collaborative multi-laboratory studies to understand the conditions for and boundaries of replication (Heinrich and Knight, 2020).

## Conclusion

There is a dearth of high quality evidence to support much of our existing clinical practice. This can be addressed by clinical trials but only if the conduct is rigorous and the quality is high. Good quality clinical trials have a research question based on PICO guidelines, follow best practice on methodological issues such as design, randomizing treatments and full blinding (participants and assessors) and choose optimal outcomes to assess the research questions in the correct timeframe and with reliability and validity. The importance of transparency and open science practices cannot be over-estimated.

## Data Availability Statement

The original contributions presented in the study are included in the article/supplementary material, further inquiries can be directed to the corresponding author.

## Author Contributions

KM proposed the topic. All authors contributed equally to draft and revised the manuscript and approved the final submission.

## Conflict of Interest

The authors declare that the research was conducted in the absence of any commercial or financial relationships that could be construed as a potential conflict of interest.

## Publisher’s Note

All claims expressed in this article are solely those of the authors and do not necessarily represent those of their affiliated organizations, or those of the publisher, the editors and the reviewers. Any product that may be evaluated in this article, or claim that may be made by its manufacturer, is not guaranteed or endorsed by the publisher.

## Footnotes

1
http://www.prisma-statement.org

2
https://www.comet-initiative.org/

3
https://www.cosmin.nl/
